# Utility of indocyanine green videoangiography with FLOW 800 analysis in brain tumour resection as a venous protection technique

**DOI:** 10.1186/s12893-022-01573-4

**Published:** 2022-04-02

**Authors:** Yue Sun, Zilan Wang, Fan Jiang, Xingyu Yang, Xin Tan, Zhouqing Chen, Yanfei Liu, Yun Zhu, Zhong Wang, Gang Chen

**Affiliations:** 1 Department of Neurosurgery, The First Affiliated Hospital of Soochow University, 188 Shizi Street, Suzhou, 215006 Jiangsu China; 2 School of Biology and Basic Medical Science, Soochow University, Suzhou, 215006 China; 3 Department of Neurology, The Affiliated Hospital of Nanjing Medical University, Suzhou Municipal Hospital, Suzhou, 215006 Jiangsu China; 4 Department of Neurosurgery, Suzhou Integrated Traditional Chinese and Western Medicine Hospital, Suzhou, 215006 Jiangsu China

**Keywords:** FLOW 800, ICG-VA, Venous preservation, Brain tumour, Neurosurgery

## Abstract

**Background:**

In regard to central nervous system tumour resection, preserving vital venous structures to avoid devastating consequences such as brain oedema and haemorrhage is important. However, in clinical practice, it is difficult to obtain clear and vivid intraoperative venous visualization and blood flow analyses.

**Methods:**

We retrospectively reviewed patients who underwent brain tumour resection with the application of indocyanine green videoangiography (ICG-VA) integrated with FLOW 800 from February 2019 to December 2020 and present our clinical cases to demonstrate the process of venous preservation. Galen, sylvian and superior cerebral veins were included in these cases.

**Results:**

Clear documentation of the veins from different venous groups was obtained via ICG-VA integrated with FLOW 800, which semiquantitatively analysed the flow dynamics. ICG-VA integrated with FLOW 800 enabled us to achieve brain tumour resection without venous injury or obstruction of venous flux.

**Conclusions:**

ICG-VA integrated with FLOW 800 is an available method for venous preservation, although further comparisons between ICG-VA integrated with FLOW 800 and other techniques of intraoperative blood flow monitoring is needed.

## Introduction

A great number of postoperative complications after resection of central nervous system (CNS) tumours are related to the failure to prevent or recognize venous problems [1]. Vital venous structures include the major dural sinuses, such as the superior sagittal sinus, deep veins, such as the vein of Galen and some dominant superficial veins, such as the vein of Labbe [2]. Iatrogenic venous injury may manifest as brain oedema, intracranial hypertension and haemorrhagic infarcts. Therefore, having a good knowledge of venous anatomy and physiology is critical.

In addition to magnetic resonance angiography (MRA), magnetic resonance venography (MRV), transcranial Doppler (TCD) and digital subtraction angiography (DSA), microscope-integrated indocyanine green videoangiography (ICG-VA) is recognized as a beneficial adjunct to determine the flow dynamics of cerebral vessels [3]. Flow dynamics are semiquantitatively analysed with FLOW 800 software integrated in the surgical microscope. Fluorescence intensities in regions of interest (ROIs) are calculated based on the average arbitrary intensity units. Then, the algorithm reconstructs colourful maps based on maximal fluorescence intensities and delay times. ICG-VA integrated with FLOW 800 is widely used in neurosurgery, especially for the evaluation of aneurysmal clipping, cerebral arteriovenous malformation resection and flow patency during bypass surgery. [4–9] Previous studies provided guidance for using ICG-VA and FLOW 800 in the venous sacrifice decision process.[10, 11] However, the use of ICG-VA and FLOW 800 for venous protection still lacks attention. Here, we report four typical cases of CNS tumours in close proximity to important venous structures. ICG-VA integrated with FLOW 800 was used before or after tumour resection in these patients to identify landmarks for the surgical approach, confirm venous patency and prevent potential complications caused by venous problems.

## Methods

This is a single-centre, retrospective study of patients with CNS tumours close to important veins who underwent microsurgical operations using ICG-VA and FLOW 800 from February 2019 to December 2020. The exclusion criteria included (a) patients allergic to ICG; (b) patients younger than 18 years old; and (c) patients cannot tolerate craniotomy. Written informed consent was obtained from all patients.

All patients underwent magnetic resonance imaging (MRI) as a routine examination before surgery to confirm tumour size and location. 3D images were preoperatively reconstructed based on contrast-enhanced MRI to clarify the relationship between the tumour and blood vessels, especially the veins. The ICG solution was prepared in advance and administered intravenously before and after tumour resection as required by the neurosurgeon.

Whereas some groups [11–13] describe the dose of 25 mg of ICG intravenously, others [14] commonly use a half dose. Here we administered 25 mg of ICG intravenously to the patients. Then, the integrated light source induced fluorescence, and INFRARED 800 (IR800) video filmed it. FLOW 800 software integrated into the surgical microscope (Release 2.21, Carl Zeiss Co., Germany) generated a colour-coded map in a timely manner for blood flow assessment via IR800 video, using red for early appearance, yellow or green for medium appearance, and blue for late appearance. Fluorescence intensity curves in ROIs were also provided by FLOW 800 software for haemodynamic analysis. When veins were confirmed to be patent after tumour resection according to the colour map and the fluorescence intensity curves, they were considered to be preserved, and no additional surgical intervention was needed.

We collected information from every patient that included age, sex, clinical manifestations, radiological manifestations that demonstrated the relaltionship of the tumour and vasculature, intraoperative findings, FLOW 800 results and prognosis.

## Results

Based on the exclusion criteria, our study included 23 patients with various clinical presentations and tumour types. The age of our patients ranged from 19 to 70 (mean age of 53) and the rate of male patients was 43%. The 23 intracranial tumours of our patients included 3 high-grade gliomas, 1 haemangioblastoma and 19 meningiomas. All tumours were completely resected. The Galen, sylvian and superior cerebral veins were involved in our study. In all patients, the tumours were in close proximity to or encased veins. ICG-VA integrated with FLOW 800 was successfully performed in all patients and all the colour mapping confirmed the patency of veins. The flow dynamics of these different venous groups were semiquantitatively analysed in 18 patients (78.2%). As in some cases, the neurosurgeon operated under the microscopic field during the ICG fluorescence process, so the fluorescence intensity curve was affected. The following four cases that show protection of the abovementioned veins are presented to show the value of ICG-VA with FLOW 800 in tumour resection surgery.

## Case presentation

### Case 1

**Fig. 1:**
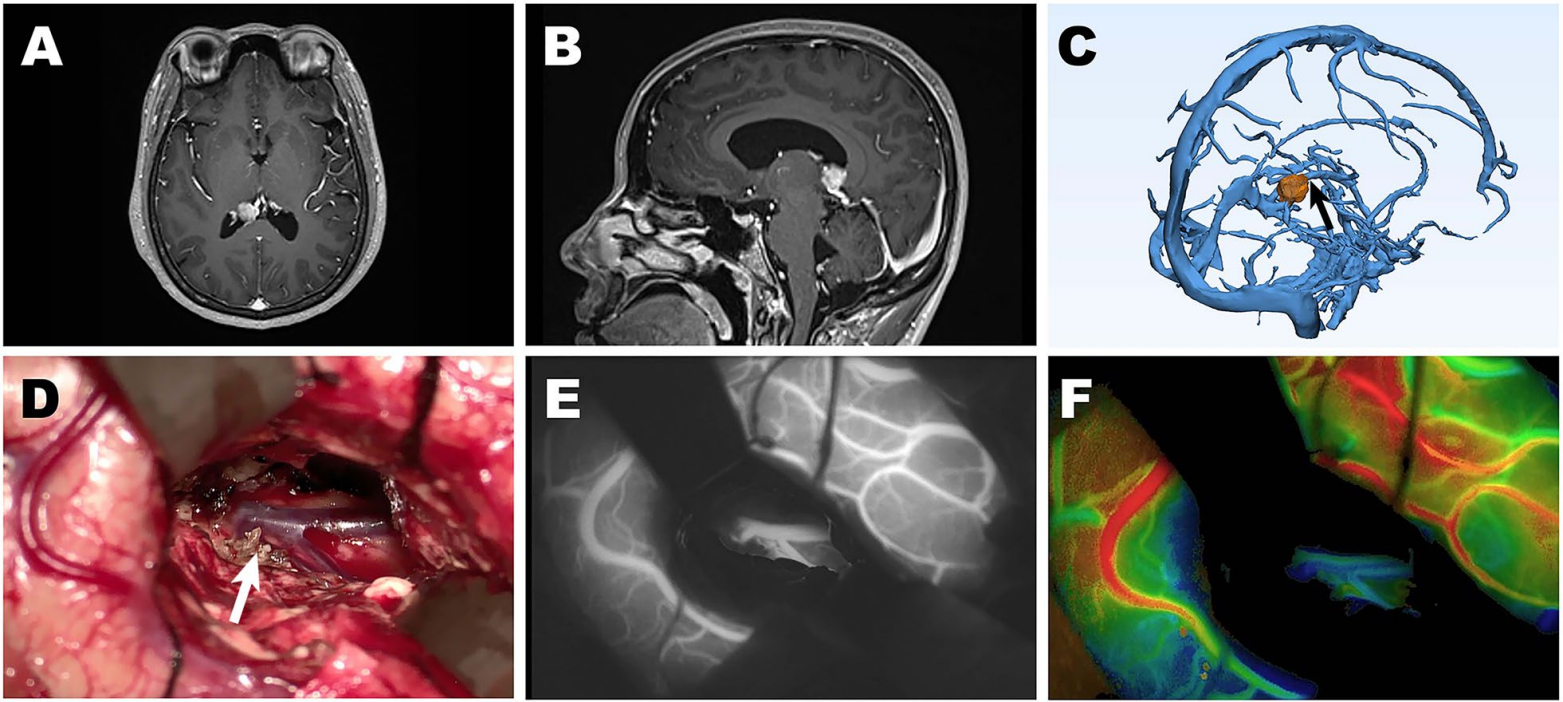
A patient with a mass located at the posterior horn of the right lateral ventricle underwent surgery. **A** T1- weighted axial and **B** T1-weighted sagittal MRI with contrast demonstrated a mass located at the posterior horn of the right lateral ventricle. **C** 3D imaging revealed the involvement of the Galen vein (black arrow) by the tumour (orange). **D** During the surgical process, the Galen vein (arrow) was preserved. **E**, **F** ICG-VA integrated with FLOW 800 clearly demonstrated provided blood flow information of the deep vein

A 30-year-old woman presented with a mass located at the posterior horn of the right lateral ventricle with no apparent clinical symptoms (Fig. 1A, B). As shown in the 3D reconstruction imaging, the mass was close to the Galen vein (Fig. 1C). Intraoperative neurosurgical navigation was used. During the surgical process (Fig. 1D), with the aid of ICG-VA integrated with FLOW 800, visualization and identification of the deep veins were achieved (Fig. 1E, F). The Galen vein was protected and remained intact, and the patient had a good prognosis.

### Case 2

**Fig. 2:**
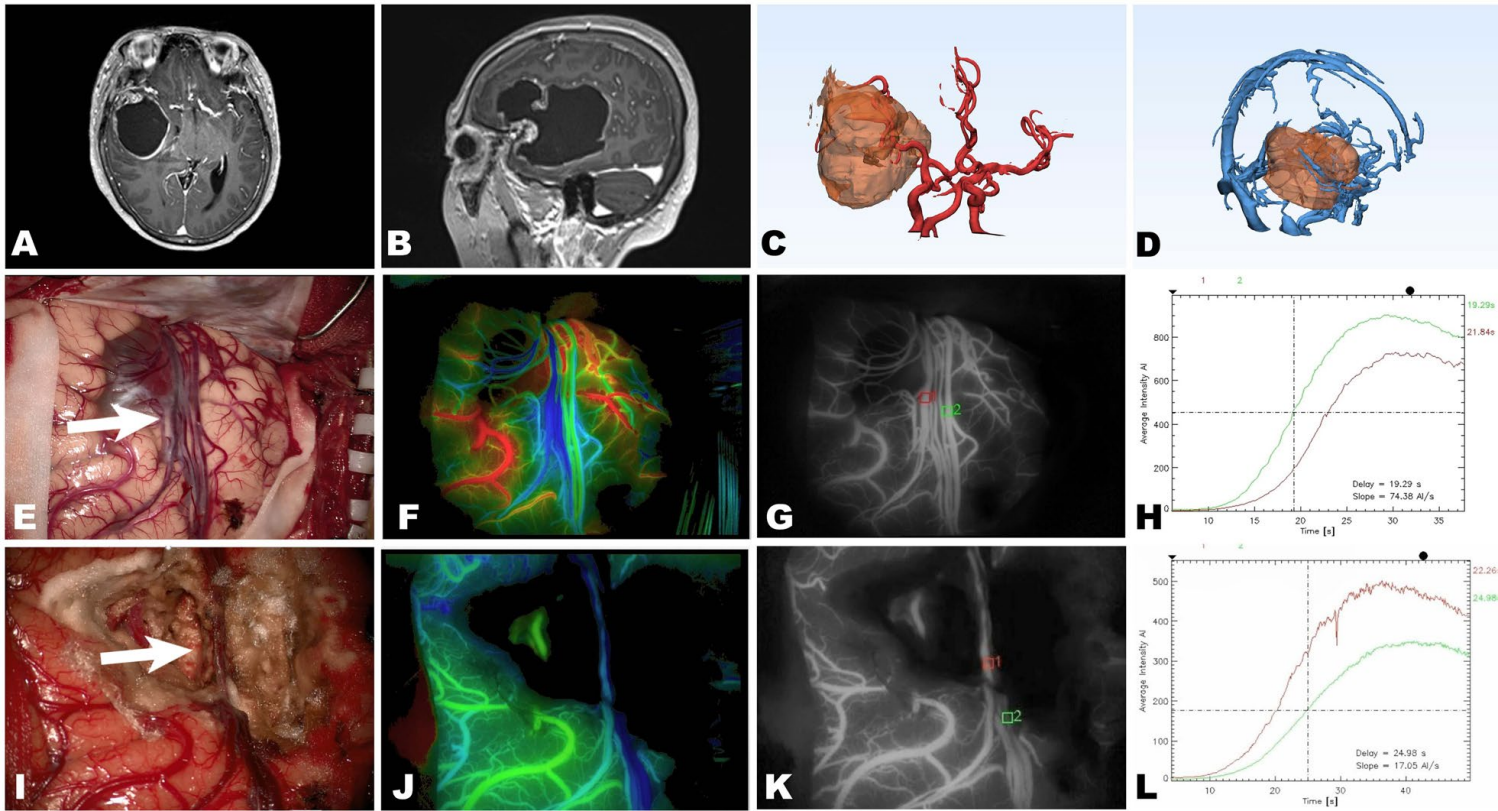
A patient with a large cystic mass in the right frontotemporal lobe underwent surgery. **A** T1-weighted axial and **B** T1-weighted sagittal MRI with contrast demonstrated a cystic mass located at the right frontotemporal lobe. **C** 3D reconstruction imaging showed the right middle cerebral artery was displaced and encased by the mass. **D** 3D reconstructed images also revealed the encased sylvian vein. **E**, **I** The mass was removed via a pterional approach and the sylvian vein (arrow) was preserved, **F**–**L** ICG-VA integrated with FLOW 800 confirmed the patency of the sylvian vein, and semiquantitatively analysed the blood flow of the protected vein

A 58-year-old woman who complained about intermittent headache showed a large cystic mass in the frontotemporal lobe. The mass was 7.7*5.4*5.3 cm in size, with midline shift and compression on the lateral ventricle (Fig. 2A, B). The 3D reconstruction imaging revealed that the right middle cerebral artery was displaced and encased by the mass (Fig. 2C), and the sylvian vein was also compressed by the mass (Fig. 2D). The mass was removed via a pterional approach (Fig. 2E, I). With the help of ICG-VA and FLOW 800, we removed the tumour while minimizing injury to the sylvian vein (Fig. 2E–G, I–K). The pre- and post-FLOW 800 analysis on the same two veins also indicated that the venous flow was almost unaffected. The patient was discharged soon after the operation without any complications.

### Case 3

**Fig. 3:**
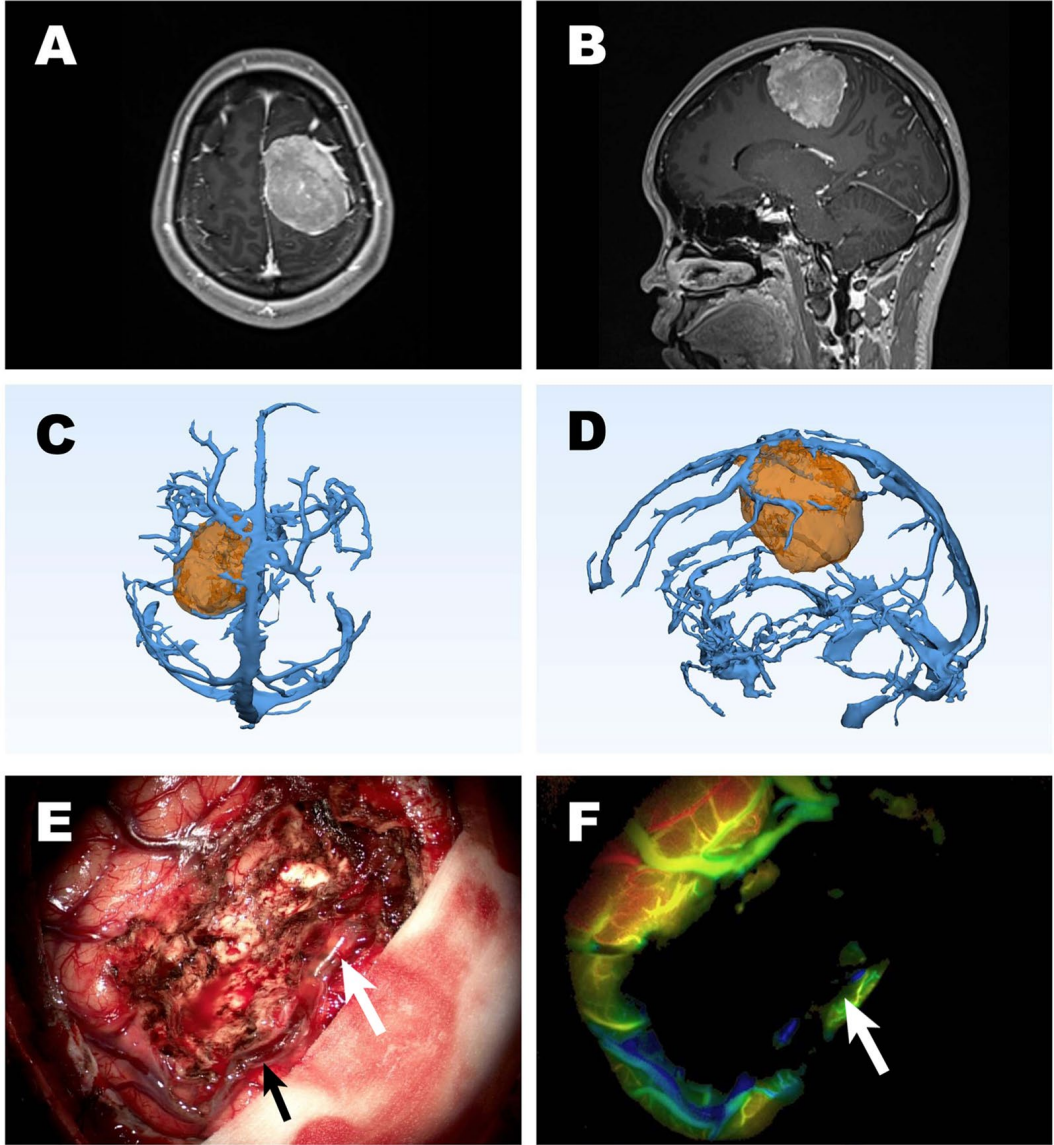
A patient with a left parietal lobe lesion underwent surgery. **A** T1- weighted axial and **B** T1-weighted sagittal MRI with contrast demonstrated a large meningioma with a significant mass effect. **C**, **D** The postcentral sulcus vein was encased and displaced by the tumour, as demonstrated on 3D reconstruction imaging. **E** The postcentral sulcus vein (black arrow) was anatomically preserved under a microscope, although **F** a hyaline thrombus (white arrow) was found based on ICG-VA with FLOW 800, and then we noticed it under the microscopic field (white arrow)

A 36-year-old woman presented with persistent headache. Magnetic resonance imaging (MRI) with contrast showed a left parietal lobe lesion that was approximately 4.5*5 cm in size (Fig. 3A, B). The lesion had a close relationship with these superior cerebral veins according to 3D reconstruction (Fig. 3C, D). The patient underwent tumour resection. The patient’s precentral- and postcentral gyrus and surrounding veins, such as the central sulcus vein and postcentral sulcus vein, were all anatomically preserved under the microscope. After administering the intravenous bolus of ICG, the veins were clearly marked, and the mapping reconstructed by the software demonstrated the blood flow condition. However, ICG-VA with FLOW 800 indicated occlusion of the postcentral sulcus vein (Fig. 3F). We then noticed a hyaline thrombus in the postcentral sulcus vein, which might have been caused by traction and compression during tumour resection (Fig. 3E). Special attention was given to postoperative management to prevent brain oedema, epilepsy and bleeding after venous infarction. The patient did well and was discharged soon after the operation.

### Case 4

**Fig. 4:**
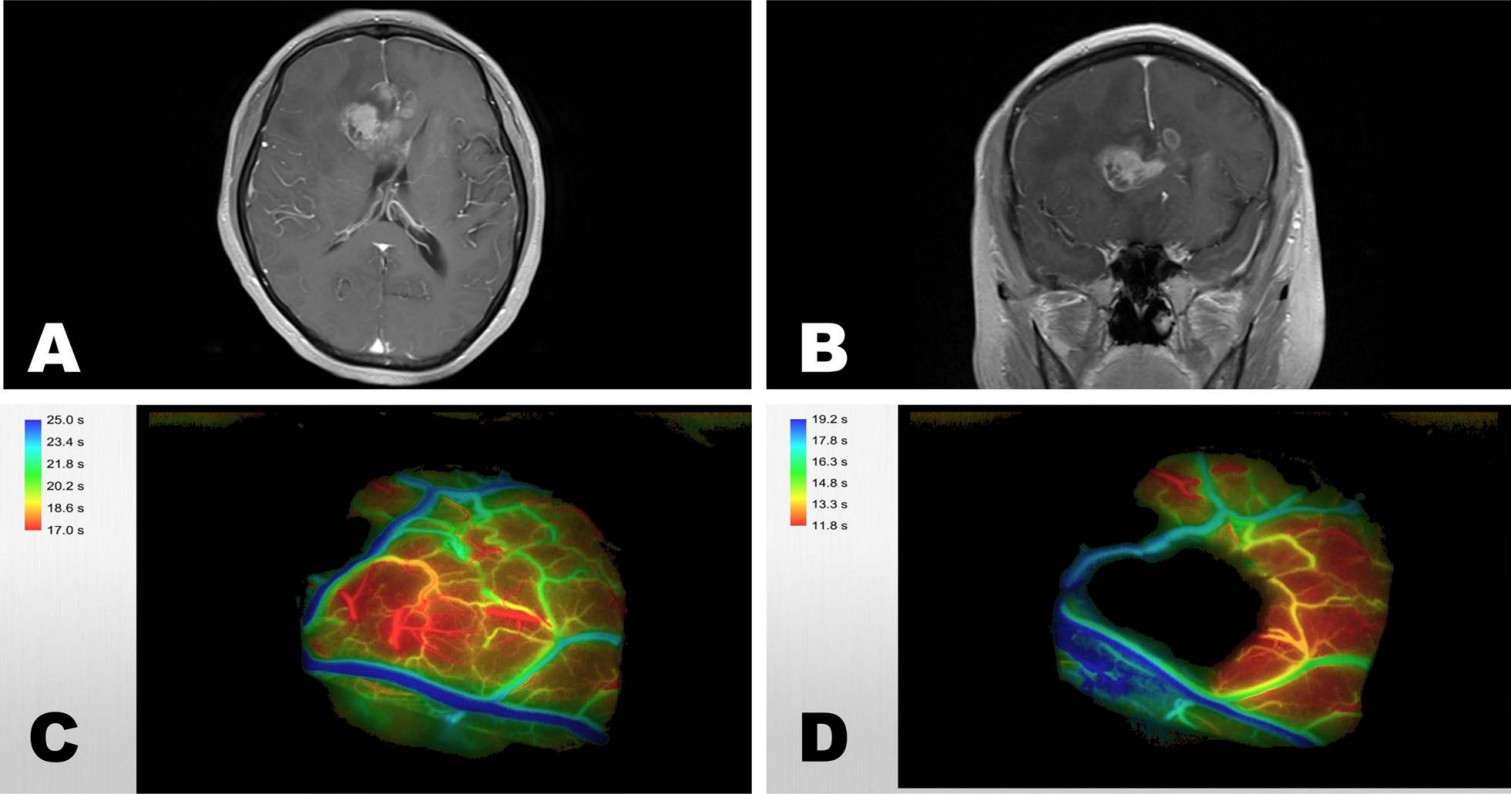
A patient with a mass on the bilateral frontal lobe and corpus callosum. **A**, **B** MRI with contrast showed a heterogeneous mass with cystic areas involving the body of the corpus callosum and extending bilaterally into the frontal lobes FLOW 800 was used to map the blood flow conditions of the two superior cerebral veins **C** before and **D** after tumour resection

A 56-year-old woman presented with a one-month history of headache and intermittent vomiting. Symptoms worsened with urinary and faecal incontinence for 20 days. MRI with contrast showed a necrotic mass with an irregular shape on the bilateral frontal lobe and corpus callosum (Fig. 4A, B). We used ICG-VA and FLOW 800 to guide the preservation of the two superior cerebral veins when performing the transcortical surgical approach to remove the tumour (Fig. 4C, D). The patient was discharged without worsening symptoms.

## Discussion

In recent decades, an increasing number of neurosurgeons have realized the importance of venous preservation because injury to or occlusion of important intracranial venous structures might lead to severe complications, such as haematoma, epilepsy, cerebral oedema, hemiplegia, and aphasia [15]. Instead of depending heavily on their own surgical experience, neurosurgeons have taken advantage of neuroimaging tools such as DSA, MRA, MRV and TCD preoperatively to analyse the vascular structures surrounding the lesion. In addition, the development of intraoperative microvascular Doppler (MVD) and ICG-VA has made the visualization of intraoperative blood flow possible. These techniques help neurosurgeons understand the dynamic flow of the venous system and how to preserve them.

Groups of veins are treated differently after neurosurgeons make particular surgical judgements, involving the consideration of whether to preserve them [16]. The superficial cerebral veins are strongly interconnected, making it acceptable to sacrifice one of them. Additionally, once the terminal ends of the sylvian vein leave the fissure and enter the sphenobasal, sphenoparietal, or cavernous sinus, they can be safely sacrificed [17]. However, to avoid devastating consequences, including contralateral hemiplegia, bridging veins known as the central group of veins, should not be stretched, injured or sacrificed. Regarding the vein of Labbe, surgeons usually preserved it to avoid venous infarction of the temporal lobe at all costs. As injury to the deep venous system can lead to diencephalic oedema, hyperpyrexia and death, preservation of deep veins such as the vein of Galen is of great concern.[18–21] However, there are no clear guidelines for venous preservation, and it has been important to make surgical judgments on a case-by-case basis with the help of imaging tools.

In patients undergoing craniotomy for tumour resection, thrombosis of the cerebral veins and sinuses occurs from time to time. The symptoms have been highly variable, including headaches, seizures and delirium, which resulted in difficulties in providing a timely diagnosis [22]. When the infarcts are associated with increased intracranial pressure, patients might die because of cerebral herniation. To clarify the formation of vein thrombosis and deliver treatment as rapidly as possible, it has been important to use ICG-VA integrated with FLOW 800 during the surgical process. A preresection survey using ICG-VA integrated with FLOW 800 helped surgeons identify the pathophysiological changes in brain veins related to the tumour and individuate landmarks for the surgical approach, while a postresection survey, as demonstrated in our patients, helped surgeons confirm venous patency to reduce the risk of postoperative complications, including venous infarction and local hypoperfusion. Compared with conventional ICG-VA, the colour maps reconstructed by FLOW 800 software enables more intuitive cerebral blood flow monitoring. And the fluorescence intensity curve in ROIs with FLOW 800 allowed surgeons to study blood flow semiquantitatively in the same vein pre-and post-operatively.

As the first to report the use of microscope-integrated quantitative analysis of ICG-VA for blood flow assessment, Kamp et al. showed great value of the maps and promoted clinical applications of FLOW 800 analysis [6]. However, few articles have reported the use of ICG-VA along with FLOW 800, especially in the field of surgery for brain tumours, and cerebral and spinal haemangioblastomas [14, 23, 24]. In the present case reports, we described our experience with using ICG-VA integrated with FLOW 800 in brain tumour resection to observe venous flux and detect the obstruction of venous reflux in a timely manner. All of our patients had a good prognosis after surgery.

There were several limitations of our study. First, we included a small number of participants. Second, the use of ICG-VA with FLOW 800 was subjective based on the experience of the surgeon. We have not identified an optimal protocol for routine use throughout the procedure. In addition, quantitative data from FLOW 800 were not systematically collected and analysed. Furthermore, long-term follow-up investigations are needed.

A comparison between ICG-VA integrated with FLOW 800 and other techniques of blood flow monitoring such as MVD and intraoperative DSA is needed to demonstrate the sensitivity and specificity. We believe that the application of ICG-VA integrated with FLOW 800 will be expanded in the future.

## Conclusion

Venous preservation during brain tumour resection can be another valuable application of ICG-VA integrated with FLOW 800. This technology can provide information on blood flow of the target vein.

## Data Availability

The data that support the findings of this study are openly available in *figshare*, reference number 10.6084/m9.figshare.19328858.

## Abbreviations

ICG-VA: Indocyanine green videoangiography
MRA: Magnetic resonance angiography
MRV: Magnetic resonance venography
TCD: Transcranial doppler
DSA: Digital subtraction angiography
MRI: Magnetic resonance imaging
CNS: Central nervous system
MVD: Microvascular Doppler
